# First Data on the Helminth Community of the Smallest Living Mammal on Earth, the Etruscan Pygmy Shrew, *Suncus etruscus* (Savi, 1822) (Eulipotyphla: Soricidae)

**DOI:** 10.3390/ani11072074

**Published:** 2021-07-12

**Authors:** María Teresa Galán-Puchades, Santiago Mas-Coma, María Adela Valero, Màrius V. Fuentes

**Affiliations:** 1 Parasite and Health Research Group, Department of Pharmacy and Pharmaceutical Technology and Parasitology, Faculty of Pharmacy, University of Valencia, 46100 Burjassot, Valencia, Spain; mario.v.fuentes@uv.es; 2 Departamento de Parasitología, Facultad de Farmacia, Universidad de Valencia, 46100 Burjassot, Valencia, Spain; s.mas.coma@uv.es (S.M.-C.); madela.valero@uv.es (M.A.V.)

**Keywords:** *Suncus etruscus*, *Mesocestoides* sp. *larvae*, *Joyeuxiella pasqualei larvae*, *Staphylocystis claudevaucheri*, *S. cerberensis*, *S. banyulsensis*, *Pseudhymenolepis* sp., *Aonchotheca* sp., helminth community

## Abstract

**Simple Summary:**

The Etruscan shrew, *Suncus etruscus*, is the smallest living mammal on Earth. Its minute size (most adults weigh 1.8–3 g with a body length of 35–48 mm) makes it extremely difficult to catch in small mammal traps. The French scientist Dr Roger Fons (1946–2016) developed a particular trapping method which allowed him to assemble the largest collection of *S. etruscus* in the world. We had the unique opportunity of studying, for the first time, the helminth community of a total of 166 individuals of the Etruscan shrew. We found six cestode species, specifically, two extraintestinal larvae, and four intestinal adult tapeworms, as well as one adult nematode species in the stomach and several nematode larvae. Neither trematode nor acanthocephalan species were detected. Approximately 50% of the individuals harbored tapeworms presenting a two-host life cycle with arthropods as intermediate hosts, a fact that is consistent with its insectivorous diet. The adult helminth community found is highly specific of this shrew whose numerous physiological adaptations due to its small size have probably influenced its helminth spectrum as well as its helminth specificity.

**Abstract:**

*Suncus etruscus* is the smallest living mammal on Earth by mass. Most adults weigh 1.8–3 g with a body length of 35–48 mm. Catching it in small mammal traps in nature is extremely difficult due to its minute size, and therefore special trapping methods must be used. We had the unique opportunity of studying, for the first time, the helminth parasites of 166 individuals of *S. etruscus*, part of the largest collection in the world, which belonged to the French scientist Dr Roger Fons (1942–2016). A total of 150 individuals were captured in the Banyuls-Cerbère area (France) and 16 in the island of Corsica (France). We found seven helminth species, specifically, the cestodes *Joyeuxiella pasqualei larvae*, *Mesocestoides* sp. *larvae*, *Staphylocystis claudevaucheri*, *S. banyulsensis*, *S. cerberensis*, and *Pseudhymenolepis* sp., and the nematodes *Aonchotheca* sp. and Nematoda gen. sp. *larvae*. Neither trematodes nor acanthocephalans were detected. We provide prevalences, infracommunity compositions, and helminth associations. The adult helminth community of *S. etruscus* seems to be highly specific, i.e., oioxenous, and linked to its insectivore diet. Due to its small size, *S. etruscus* has undergone numerous physiological adaptations that have probably influenced its helminth spectrum as well as its helminth specificity.

## 1. Introduction

**Figure 1:**
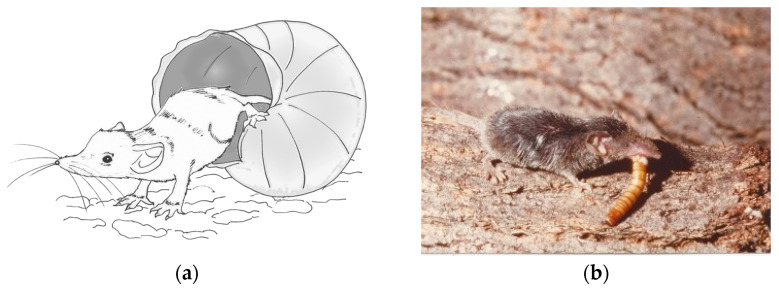
*Suncus etruscus*: (**a**) Comparative size of the smallest living mammal on Earth (illustration by Angela Debenedetti, PhD); (**b**) An individual of the Etruscan shrew ingesting a yellow mealworm (*Tenebrio molitor*) larva (R. Fons photo collection).

*Suncus etruscus,* the Etruscan shrew, also known as the Etruscan pygmy shrew, the Pygmy white-toothed shrew, or the Savi’s pygmy shrew, is, by mass, the smallest living mammal on Earth. Most adults weigh 1.8–3 g with a body length of 35–48 mm, being 20 times lighter than the average adult mouse [1] (Figure 1). This miniscule mammal species is widespread throughout Southern Europe, Northern Africa, and Central Asia. *Suncus etruscus* has indeed one of the largest distribution ranges among insectivore mammals encompassing the territory from the Iberian Peninsula to Kalimantan (Borneo) Island (Indonesia), where they occupy abandoned olive groves and vineyards with old dry stone walls and stone-piles as well as low maquis shrubs and Mediterranean oak and pine forests [2]. Recently, the Etruscan shrew has also been detected in Russia [3].

Despite its large geographical distribution, only a small number of individuals of this species have been caught in the world, since catching *S. etruscus* in small mammal traps in nature is extremely difficult due to its minute size, and therefore special trapping methods must be used. In fact, its existence was only known thanks to the findings of its predator (the barn owl *Tyto alba*) pellets [4].

Roger Fons (1942–2016), Directeur de Recherche at the French CNRS (Centre National de la Recherche Scientifique), was the only specialist of the Etruscan pygmy shrew in the world, carrying out his scientific activity at Laboratoire Arago (University Pierre et Marie Curie, Paris VI, Banyuls-sur Mer, France). He published numerous scientific articles dealing with *S. etruscus,* as for instance those concerning trapping and breeding methods [4,5], morphology [6,7,8,9,10,11], evolution and systematics [12], biology/ecology [5,6,13,14,15,16,17], ethology [14,18,19,20], and physiology [14,21,22,23,24,25,26,27,28,29,30], among others.

Due to our, not only scientific collaboration, but also friendship with Dr Fons, we had the unique opportunity of studying, for the first time, the helminth parasites of *S. etruscus*, since he owned the largest collection of this shrew in the world. In fact, in the 1980s we jointly published the finding of three new intestinal cestodes, namely the hymenolepidids *Hymenolepis claudevaucheri* [31], *H. cerberensis* [32], and *H. banyulsensis* [33].

In addition to the three new tapeworm species, we found some other helminths in the studied individuals. Herein, we present the first data on the global helminth community of the Etruscan shrew, and we would like this article to be a tribute to Dr Fons, who owed his outstanding and well-deserved career to his hard work, his ability to solve technical problems, and the passion that drove him on.

## 2. Materials and Methods

### 2.1. Study Areas and Hosts

**Figure 2:**
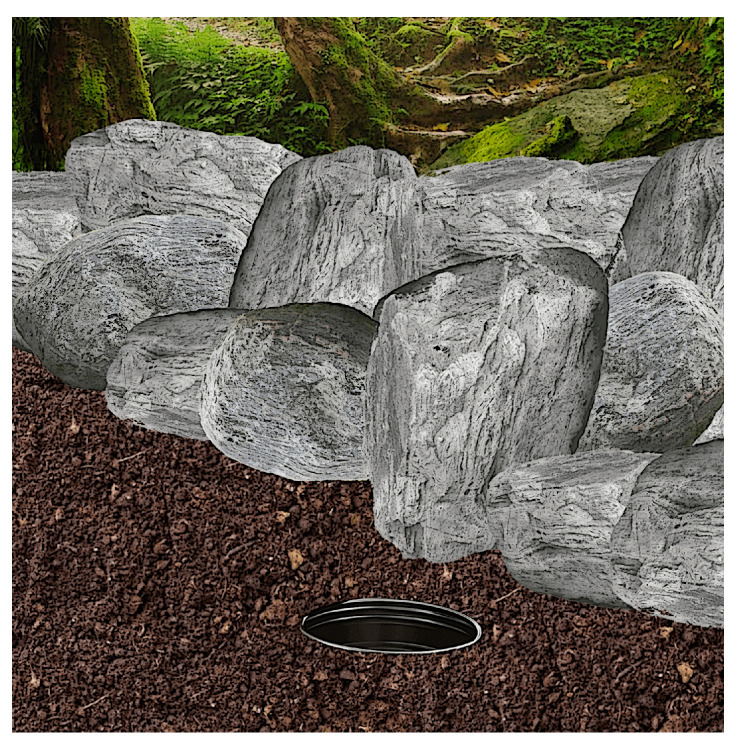
Interception trap buried to ground level at the bottom of a dry stone wall (illustration by Angela Debenedetti, PhD).

A total of 166 *S. etruscus* (97 males and 69 females) was examined in order to study their helminth parasites. Concerning the study areas, 150 individuals (86 males and 64 females) were trapped in the rural areas of the municipalities of Banyuls-sur-Mer and Cerbère (Pyrénées-Orientales department, France) and 16 individuals (11 males and five females) were captured in the Island of Corsica (France). Considering the small size of *S. etruscus*, conventional traps do not work. Dr Fons, therefore, designed, what he called “interception traps” (pitfall traps), consisting in metal boxes (25 cm high, 15 cm wide) perforated at the bottom (to prevent the accumulation of water) and buried in the ground-to-ground level. The traps were placed mainly in three types of biotopes, i.e., at the bottom of dry stone walls (not cemented) of abandoned vineyards, olive terraces reclaimed by xerophilic vegetation, and those placed in maquis shrubs. Traps do not contain any kind of bait to prevent ants from destroying the preys (Figure 2).

Not only can *S. etruscus* be captured by means of these traps but also other Soricidae like *Crocidura russula* or *C. suaveolens*. Likewise, the invertebrate fauna of the area, susceptible to being part of the diet of these insectivores, can also be caught up.

Interception traps were checked up on a daily basis, and live Etruscan shrews were kept in captivity by Dr Fons. Most of the animals found dead in the traps were preserved in 85% ethanol. We also had the opportunity of studying some individuals of *S. etruscus* shortly after their death in the interception traps placed in the Banyuls/Cerbère area along our long-lasting collaboration with Dr Fons.

The animal weight could be determined only in the 31 specimens that were not preserved in ethanol. This weight varied between 1.3–2.7 g (1.8 g).

No ethical approval was required at the time Dr Fons conducted his surveys, i.e., in the nineteen sixties–eighties.

### 2.2. Parasitological Techniques

*Suncus etruscus* individuals were dissected in order to extract the internal organs at Laboratoire Arago. Due to the small size of *S. etruscus*, the specimens were dissected under a stereomicroscope (Meiji Techno^®^ EMT Stereo Microscope). The organs (gastrointestinal tract, lungs, liver, spleen, urinary system) were place in saline in Petri dishes and were stereomicroscopically examined. The helminths found (cestodes and nematodes) were placed in 70% ethanol until their subsequent study at Department of Parasitology of University of Valencia.

Following conventional helminthological techniques, cestodes were stained with alcoholic hydrochloric carmine for 24 h. Subsequently, the helminths were partially destained with acidified alcohol, dehydrated in an alcohol series, cleared with xylene, and mounted in Canada balsam between slide and cover slip. Prior to the staining process, in the case of adult cestode stages, the last gravid proglottids were separated in order to carry out the morphological study of the tapeworm eggs, since the staining and dehydration procedures tend to alter the egg morphology. These last gravid proglottids were placed on a slide in a drop of lactophenol, a clearing fluid, and were subsequently broken in order to liberate the eggs. Those tapeworms that were collected alive were previously relaxed and fixed by shaking them in hot 70% ethanol.

Nematodes were studied by direct examination between slide and cover slip with lactophenol.

### 2.3. Statistical Analysis

The number of parasitized shrews, the overall prevalence of infection, as well as the prevalence of each helminth species, were analyzed. Standard non-parametric tests were applied to analyze the influence of host sex, season, and type of biotope (abandoned vineyards, olive terraces, and maquis shrubs) on the prevalence of the helminth parasites by Binary Logistic Regression (BRL). Positive or negative helminth associations were calculated by means of a chi-squared test (χ^2^). Statistical significance was established at *p* < 0.05. The IBM SPSS Statistics 26 for Windows software package was used for statistical analysis.

## 3. Results

**Figure 3:**
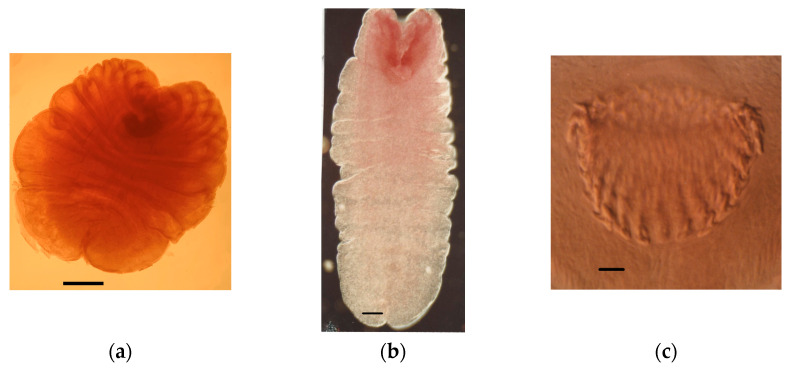
Extraintestinal metacestodes of *S. etruscus*: (**a**) tetrathyridium of *Mesocestoides* sp.; (**b**) cysticercoid of *Joyeuxiella pasqualei*; (**c**) detail of the rows of hooks of the *J. pasqualei* metacestode. Scale bars: (**a**), 400 μm; (**b**), 150 μm; (**c**), 20 μm.

**Figure 4:**
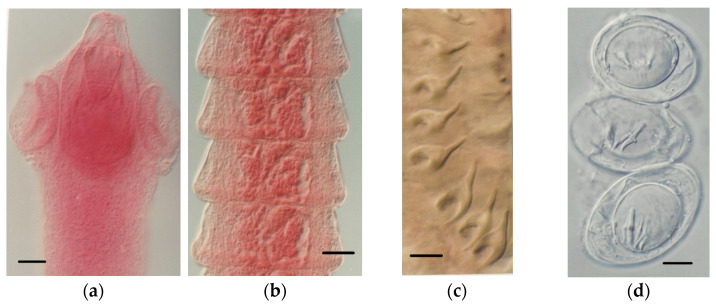
*S. claudevaucheri*: (**a**) armed scolex; (**b**) postmature proglottids. *S. cerberensis*: (**c**) rostellar hooks; (**d**) eggs. Scale bars: (**a**), 30 μm; (**b**), 80 μm; (**c**,**d**), 10 μm.

**Figure 5:**
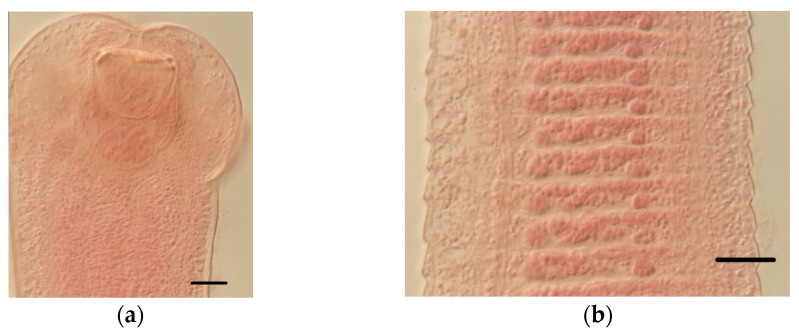
*S. banyulsensis*: (**a**) armed scolex; (**b**) premature proglottids. Scale bars: (**a**,**b**), 30 μm.

**Figure 6:**
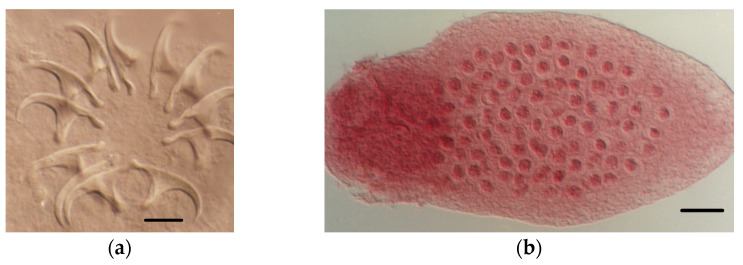
*Pseudhymenolepis* sp.: (**a**) rostellar hooks; (**b**) gravid proglottids containing uniovular capsules. Scale bars: (**a**,**b**), 10 μm.

Eighty-four out of the 166 *S. etruscus* examined (50.60%) were parasitized. Table 1 details the helminth community found, made up of seven helminth species, including the extra-intestinal larval stages (metacestodes) of two cestodes, *Mesocestoides* sp. Vaillant, 1863 *larvae* and *Joyeuxiella pasqualei* (Diamare, 1983) *larvae* (Figure 3), the intestinal adult stages of four more tapeworms, *Staphylocystis claudevaucheri* (Mas-Coma, Fons, Galán-Puchades, and Valero, 1984)*, S. cerberensis* (Mas-Coma, Fons, Galán-Puchades, and Valero, 1986)*, S. banyulsensis* (Mas-Coma, Fons, Galán-Puchades, and Valero, 1986) and *Pseudhymenolepis* Joyeux and Baer, 1935 sp. (Figure 4, Figure 5 and Figure 6), and one stomachal nematode species, *Aonchotheca* López-Neyra, 1947 sp. In addition to these helminths, fragments of tapeworm strobilae consistent with cestodes belonging to the hymenolepididae family were detected in nine *S. etruscus*. However, due to their bad preservation, it was impossible to classify them at genus or species level. Likewise, in six individuals, young larval stages of nematodes found in the stomach or abdominal cavity were classified as Nematoda gen. sp. *larvae* due to the lack of distinctive morphological features.

Half of the *S. etruscus* population (50.60%) was parasitized. Cestodes, with almost 50% of parasitation, were clearly the most prevalent parasites in the helminth community, whilst nematodes were scarcely distributed in the host population. Only 5 individuals (3.01%) harbored not only cestodes but also nematodes. Neither trematodes nor acanthocephalans were detected.

*Pseudhymenolepis* sp. (28.31%) was the most prevalent cestode followed by *S. claudevaucheri* (18.67%).

According to sex, 52 out of the 97 males were parasitized (53.61%) and 32 out of the 69 females (46.38%). There were no statistical differences when considering host sex, season, or biotope of capture.

Concerning infracommunities (helminths of different species in the same host- [34]), 58 *S. etruscus* harbored only one helminth species (64.29% among the parasitized), 20 harbored two species (23.81%), five harbored three species (5.95%), and one *S. etruscus* (1.19%) captured in the Banyuls/Cerbère area was parasitized by the four intestinal adult tapeworm species.

Table 2 summarizes the helminth communities of the Etruscan shrew according to the studied area, i.e., Banyuls/Cerbère and Corsica.

The statistical comparison between the studied areas revealed no differences in the overall prevalence of helminths, although the low number of *S. etruscus* studied in Corsica limits the statistical analysis. This very much smaller number of *S. etruscus* studied in Corsica compared to those examined in Banyuls/Cerbère (16 vs. 150), understandably limits any comparative analysis between these two areas. Therefore, the poorer helminth community of *S. etruscus* in the island (lack of certain cestodes or no nematodes) is probably due to this fact. In spite of these different numbers, *Pseudhymenolepis* sp. is the most prevalent helminth in both areas.

Three positive helminth associations, i.e., *S. claudevaucheri-S. banyulsensis* (χ^2^ = 7755; *p* = 0.0054), *S. claudevaucheri-S. cerberensis* (χ^2^ = 15.297; *p* < 0.0001), and *S. banyulsensis-S. cerberensis* (χ^2^ = 11.534; *p* = 0.0007) were found.

## 4. Discussion

### 4.1. Helminth Populations

#### 4.1.1. Tapeworm Metacestodes

According to our results, *S. etruscus* represents a new host for the metacestodes of *Mesocestoides* sp. and *Joyeuxiella pasqualei.* In contrast with the remaining Cyclophyllidean cestodes, the life cycles of both tapeworms are not completely understood yet. Small mammals could act as (second?) intermediate hosts or paratenic hosts in the two life cycles [35].

- *Mesocestoides* sp.

**Figure 7:**
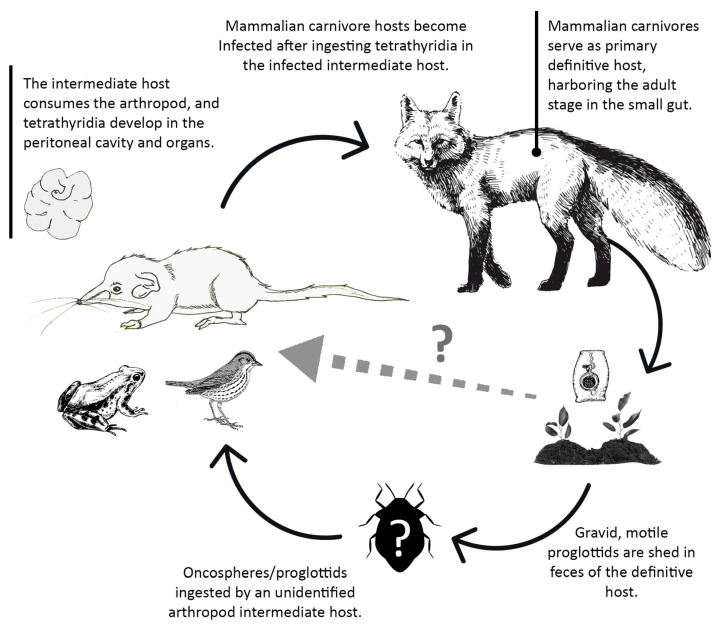
Partially known *Mesocestoides* life cycle (illustration by Angela Debenedetti, PhD).

Adult stages of tapeworms of the genus *Mesocestoides* infect a variety of terrestrial mammalian carnivores. In its life cycle, there seems to be a missing link in between the definitive hosts and the hosts harboring the metacestode, known as tetrathyridium (Figure 7). The host specificity of tetrathyridia is remarkably low, having been found in a large variety of animals such as mammals, birds, reptiles, and amphibians [36]. The repeated failures in trying to infect vertebrates with *Mesocestoides* eggs led to the assumption that there might be an invertebrate first intermediate host in this life cycle [37]. Various authors have proposed terrestrial arthropods such as dung beetles, ants, roaches, and mites as potential first intermediate hosts [38]. However, recently, it has been suggested that *Mesocestoides* might develop through a simple two-host (diheteroxenous) life cycle rather than an obligate three-host cycle (triheteroxenous), only using vertebrates as the intermediate host [39].

**Figure 8:**
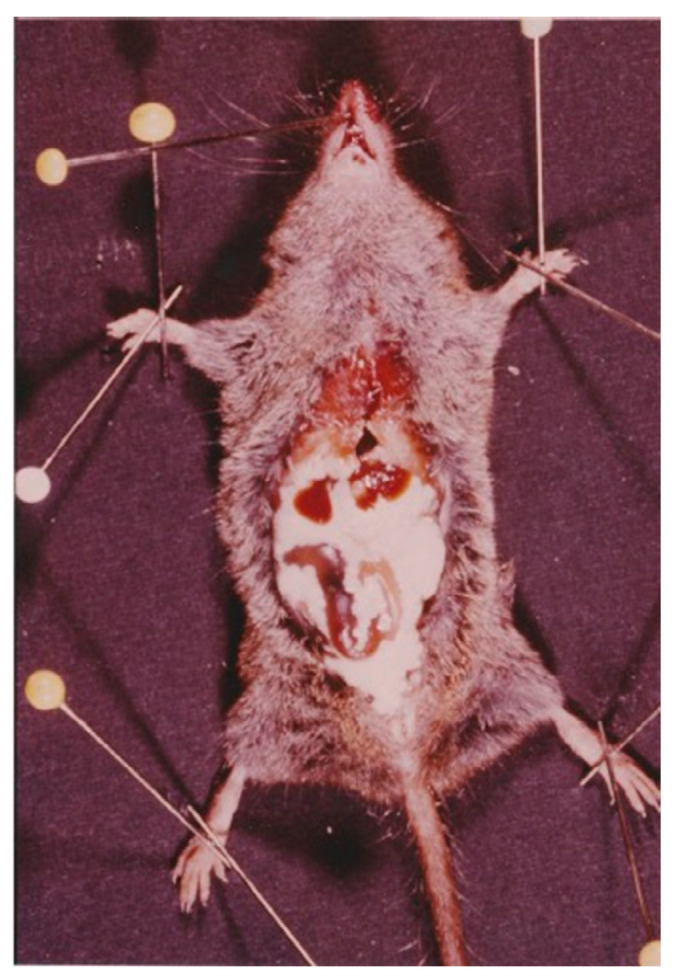
Mass-infection by *Mesocestoides* tetrathyridia in an individual of *Crocidura suaveolens* captured in the island of Corsica (France).

Five *Mesocestoides* tetrathyridia were found in the abdominal cavity of one individual of *S. etruscus* in the island of Corsica. No parasitized Etruscan shrew with this metacestode was found in the Banyuls/Cerbère area. Likewise, neither was tetrathyridia found in 69 *Crocidura russula* (the greater white-toothed shrew) studied in the same area [40]. However, 23 out of 141 (16.30%) *Crocidura suaveolens* (the Lesser white-toothed shrew) from Corsica harbored tetrathyridia of *Mesocestoides* sp. [36]. Mass-infections were frequently detected in the positive shrews [40] (Figure 8). These cases in which up to hundreds of metacestodes were found in several *C. suaveolens* in Corsica could be a consequence of the asexual reproduction by tetrathyridia [41,42] or, assuming a two-host life cycle, a consequence of the lesser shrew having ingested the intact proglottids containing the parauterine organ which protects the high number of oncospheres included inside. Therefore, both of these reproductive strategies of *Mesocestoides* are probably lethal or very harmful for *S. etruscus* considering its minuscule size (tetrathyridia of up to 4.01 mm long × 3.85 mm wide were found in the infected *S. etruscus* from Corsica) (Figure 3a). As a consequence, parasitized Etruscan shrews would probably die or would be very easily preyed upon by the definitive host, which could explain why only one of 166 *S. etruscus* was found parasitized by *Mesocesoides* tetrathyridia. However, the absence of *Mesocestoides* tetrathyridia in the Etruscan shrew could also be a consequence, assuming a three-host life cycle, of the *S. etruscus* diet, which would normally not include the arthropod intermediate host.

In the *Mesocestoides* life cycle, *S. etruscus* is probably an intermediate host instead of a paratenic one. The Etruscan shrew could become infected through the ingestion of either an infected arthropod (in the case of a three-host life cycle), or through the ingestion of gravid proglottids (in the case of a two-host life cycle). Although *S. etruscus* often kill insects of up to half their size, despite their hunger, they are frightened by and never attack vertebrates such as mice, birds, large reptiles, or amphibians that could harbor the tetrathyridia metacestodes [18]. However, when faced with a lack of nourishment, *S. etruscus*, just like other Soricidae, turns cannibal [18]. Therefore, *S. etruscus* could also act as paratenic host just in case the Etruscan shrew devoured an infected individual of its same species.

- *Joyeuxiella pasqualei*

**Figure 9:**
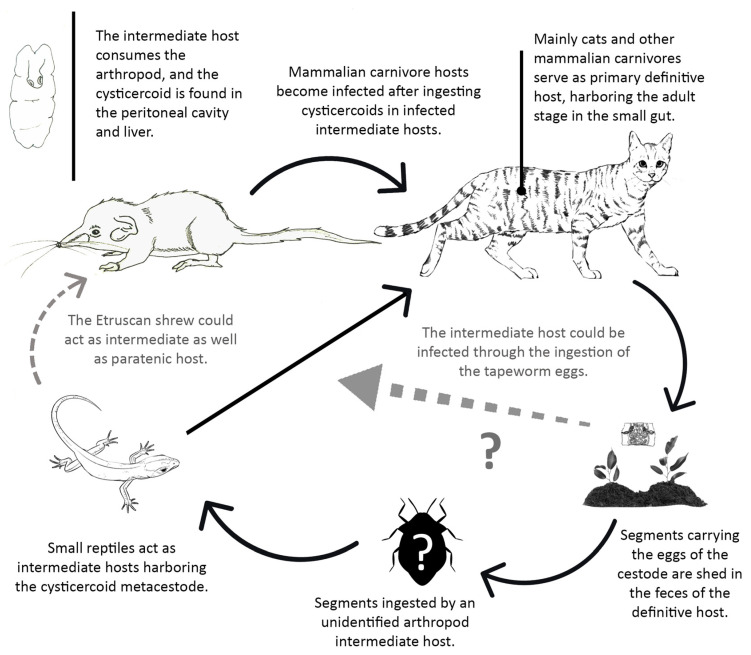
Partially known *Joyeuxiella pasqualei* life cycle (illustration by Angela Debenedetti, PhD).

The definitive hosts of the intestinal adult stages of *J. pasqualei* are mainly domestic cats, but the tapeworm has also been found to infect dogs, wolves, and servals [43]. Small reptiles, mainly lizards (*Tarentola mauritanica*, the common wall gecko, *Hemidactylus frenatus*, the house gecko and other similar reptiles) act as intermediate hosts which contain the cysticercoid metacestode that is found in the peritoneal cavity and liver (Figure 9). Sporadically, the cysticercoid has also been found in small mammals like the field mouse, *Apodemus sylvaticus* [44] and in the shrews *Crocidura russula* [44] and *C. suaveolens* [45]. Concerning its life cycle, due to the failure in infecting wall geckos with segments carrying the eggs, the existence of an insect first intermediate host was suggested [46]. However, laboratory experiments trying to infect insects like mealworms, cockroaches, and crickets with *J. pasqualei* eggs have failed so far [45].

In the liver of two individuals of *S. etruscus* captured in the Banyuls/Cerbère area, we found three small metacestodes of around 0.9–1.5 mm long and 0.4–0.7 mm wide (Figure 3b). No cysticercoid of this tapeworm was found in the 69 *C. russula* studied in the same area [40].

In the *J. pasqualei* life cycle, *S. etruscus* probably plays the role of second intermediate host (in the case of a three-host life cycle), becoming infected through the ingestion of a hypothetical insect first intermediate host. Assuming a two-host life cycle, *S. etruscus* would act as intermediate host becoming infected through the ingestion of the tapeworm eggs. Due to its minuscule size, becoming infected by means of the depredation of a gecko-the prey being larger than the predator-seems unlikely. However, Dr Fons observed that, in captivity, *S. etruscus* sometimes attacks the common wall gecko [18]. Therefore, the Etruscan shrew could act as intermediate as well as paratenic host in the life cycle of *J. pasqualei*.

Just as in the case of the *Mesocesoides* life cycle, *S. etruscus* could also act as paratenic host of *J. pasqualei* by means of cannibalism.

#### 4.1.2. Tapeworm Adult Stages

- *Staphylocystis* Species

The three intestinal adult tapeworms found in *S. etruscus*, namely *Hymenolepis claudevaucheri*, *H. cerberensis*, and *H. banyulsensis*, were described as new cestode species in the 1980s [31,32,33]. Posteriorly, and according to the key to identify to generic level cestodes of the family Hymenolepididae, published in 1994 [47], the species were reallocated to the genus *Staphylocystis*, since the three species present an armed rostellum. According to the aforementioned key, *Hymenolepis* includes species with unarmed rostella.

These intestinal hymenolepidids clearly show morphological similarities with those of the genus *Crocidura*, i.e., they all have a scolex with a well-developed rostellum inside a well-marked sheath and armed with a typical row of hooks. In addition, no representative of tapeworms belonging to the family Arostrilepididae (hymenolepidids with unarmed scolex and without rostellum, present in shrew of the subfamily Soricinae [48]) has been found in *S. etruscus*.

As far as we know, these three *Staphylocystis* species have not been found in any other small mammal in the world so far, thus indicating that they are specific of *S. etruscus*. In fact, we analyzed 662 *Crocidura russula* and 171 *C. suaveolens* from the same geographical areas in which these two shrews coexist with *S. etruscus* in southern Europe (both, in different islands and on the mainland) without finding them [40].

**Figure 10:**
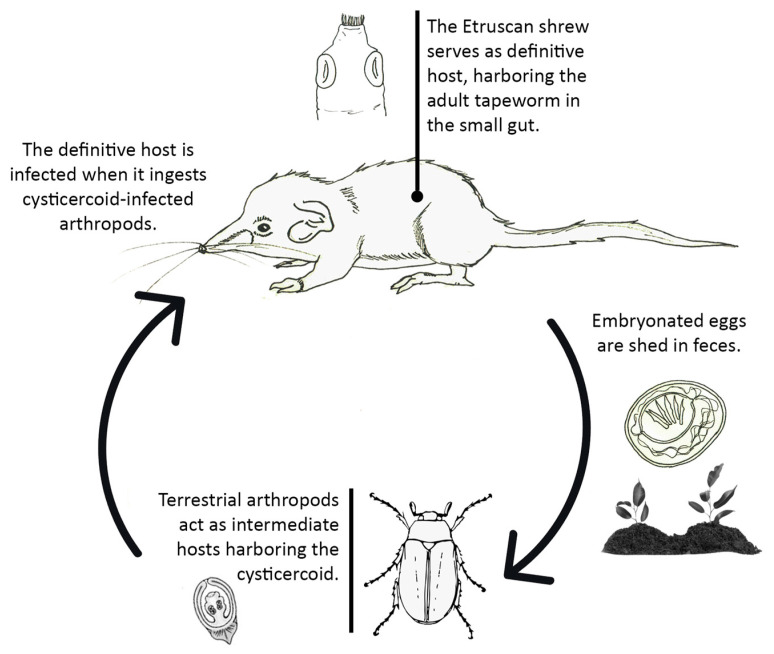
Probable life cycle of the cestode species of the family Hymenolepididae infecting *S. etruscus* (illustration by Angela Debenedetti, PhD).

Just as for the rest of cestodes of the Hymenolepididae family, a two-host life cycle with terrestrial arthropods acting as intermediate hosts harboring the cysticercoid is assumed for these tapeworms, for which the Etruscan shrew acts as definitive host (Figure 10). The positive associations found among some of these tapeworms suggest either that some of the species could share a common intermediate host, or that different arthropods intermediate hosts are commonly included in the *S. etruscus* diet.

- *Pseudhymenolepis* sp.

Almost 56% of the parasitized *S. etruscus* harbored this tapeworm, thus becoming the most prevalent parasite of its helminth community.

The peculiar cestodes of this genus have a hyperapolitic strobila (proglottids detach from the posterior part of the strobila when they are immature and live separately in the intestine) and the uterus breaks down into uniovular capsules. This genus parasitizes exclusively white-toothed shrews (subfamily Crocidurinae), specifically of the genera *Crocidura*, *Diplomesodon*, and *Suncus.*

Currently, *Pseudhymenolepis* species of the genus *Crocidura* include: *P. redonica* Joyeux and Baer, 1935 (*C. russula*, Europe), *P. eburnea* Hunkeler, 1970 (*C. jouvenetae*, Africa), *P. papillosa* Hunkeler, 1970 (*C. flavescens*, *C. jouvenetae*, *C. poensis*, Africa), *P. graeca* Vaucher, 1984 (*C. suaveolens*, Greece), *P. nepalensis* Sawada and Koyasu, 1991 (*C. suaveolens*, Kazakhstan), *P. japonica* Sawada and Harada, 1991 (*C. dsinezumi*, Japan), *P. crociduri* Velikanov, 1997 (*C. suaveolens*, Turkmenistan), and *P. spasskii* Velikanov, 1997 (*C. suaveolens*, Turkmenistan) [49,50,51,52,53,54].

*Pseudhymenolepis turkestanica* Tkach and Velikanov, 1991 (Middle Asia) is specific of the only living representative of the genus *Diplomesodon*, the piebald shrew *D. pulchellum* [55].

Regarding *Pseudhymenolepis* of the genus *Suncus,* those reported in *S. murinus* in different Asian countries are: *P. solitaria* (Meggit, 1927); *P. eisenbergi* Cruz and Sanmugasunderam, 1971; *P. guptai* Gupta and Singh, 1988; *P. lucknowensis* Gupta and Savita, 1989; and *P. nepalensis* Sawada and Koyasu, 1991 [51,56,57]. *Pseudhymenolepis suncusi* Gupta and Sinha, 1984, and *P. guptai* were also reported in *S. striatus* in Asian countries [58,59].

*P. eisenbergi* as well as *P. suncusi* and *P. guptai* are not hyperapolitic cestodes. Therefore, according to the aforementioned key to classify cestodes [47], these species do not belong to the *Pseudhymenolepis* genus. *P. lucknowensis* (India) is named in a paper by Sawada and Ohono [56], however, we have not found its original description in the literature to find out whether it is, or not, a hyperapolitic tapeworm.

In accordance with this distribution among Crocidurinae shrews, the species belonging to the *Pseudhymenolepis* genus show a high degree of specificity at host genus level. There is only one exception, the presence of *P. nepalensis*, a parasite of *S. murinus* in *C. suaveolens.* The finding was published in a congress abstract [60], however, it was not endorsed by any subsequent scientific article, so there may be a reasonable doubt about its specific identification.

The morphological characteristics of the *Pseudhymenolepis* species found in *S. etruscus* in both study areas, substantially differ from those of the currently known *Pseudhymenolepis* species. The number, size, and hook morphology (Figure 6a), as well as the size and morphology of the gravid proglottids (number of uniovular uterine capsules) (Figure 6b) allow the morphological distinction of this species. Therefore, based only on morphology, a new species could be proposed. However, we also found in four out of 13 (30.76%) individuals of *C. suaveolens* from the island of Porquerolles (Iles d’Hyères, France, in which the lesser white-toothed shrew coexists with *S. etruscus*), several proglottids and scolices of a species of *Pseudhymenolepis* similar to those found in *S. etruscus* [40]. Consequently, pending molecular studies of nuclear ribosomal RNA (complete ITS region and partial 28 S region) are needed to support the status of the *Pseudhymenolepis* found, both in *S. etruscus* and *C. suaveolens*.

Regarding the *Pseudhymenolepis* life cycle, its cysticercoids have been found in the flea *Ctenophtalmus arvernus* [61] and in the arachnid *Phalangium opilio*, the most widespread species of harvestman (opiliones) in the world [62]. No fleas have ever been found in the Etruscan pygmy shrew, probably due to its distinctly short and fine fur [63]. Therefore, other arthropods than fleas would have to act as intermediate hosts of the species of *Pseudhymenolepis* parasitizing, at least, *S. etruscus* (Figure 10). Considering that this tapeworm is the most prevalent parasite of the *S. etruscus* helminth community, the arthropod intermediate host must be a staple, more than a sporadic one, in its diet.

#### 4.1.3. Nematodes

Herein, we report the first findings of nematode larvae and the adult stages of *Aonchotheca* sp. in *S. etruscus*.

Two adult nematode specimens, a male and a female, were found in the stomach of two Etruscan shrews trapped in the Banyuls/Cerbère area (prevalence 1.33%, Table 2). Based on their morphological characteristics, both, male and female, were classified as belonging to the genus *Aonchotheca* Lopez-Neyra, 1947 *sensu* Moravec (1982) [64]. This genus includes capillariid species with male worms having lateral alae, a caudal bursa, a spicule, and a non-spiny cirrus.

*Aonchotheca minuta* (Chen, 1837) Moravec 1982, is the only species of the genus reported in a white toothed-shrew of the genus *Suncus*, in particular, in *S. murinus* in Asian countries. In different species of the genus *Crocidura*, the following have been reported: *A. africana* (Khalil, 1977) Mas-Coma and Galán-Puchades, 1985 (*C. fumosa schistacea*, Tanzania), *A. helvetica* Mas-Coma and Galán-Puchades, 1985 (*C. russula*, Switzerland), *A. europea* Mas-Coma and Galán-Puchades, 1985 (*C. russula*, southern Europe), *Aonchotheca* sp. I Mas-Coma and Galán-Puchades, 1985 (*C. russula*, Belgium), *Aonchotheca* sp. II Mas-Coma and Galán-Puchades, 1985 (*C. leucodon*, Bulgaria), and *A. crociduri* Asakawa, Kamiya, and Ohbayashi, 1988 (*C. dsinezumi dsinezumi,* Japan) [65,66].

Although the genus *Aonchotheca* has host specificity [65], the same species have sporadically been reported in other mammalian families [67]. In the case of the *Aonchotheca* specimens of *S. etruscus,* due to the scarcity of the material obtained, specific identification was not possible. Therefore, we were not able to find out if this species of *Anchotheca* is specific of the Etruscan shrew and, consequently, it would be a new species, or if *S. etruscus* and *C. russula* share the species *A. europaea,* which was found in the greater white-toothed shrew trapped in the same study area (unpublished data).

The life cycle of the members of the genus *Aonchotheca* is known for just a few species; they are diheteroxenous, with an invertebrate as intermediate host harboring the infective third-stage larvae which is acquired by the definitive host, *S. etruscus* in this case, through the ingestion of the intermediate host [68].

### 4.2. Helminth Community

According to our results, the helminth community of the 166 individuals of *S. etruscus* examined is exclusively made up of cestodes and nematodes. The species richness found, without considering the undetermined nematode larvae, consists of 7 helminth parasites. Trematodes and acantocephalans were absent and, although present, the nematode spectrum was remarkably poor.

Concerning the lack of trematode species in *S. etruscus*, we found different flukes in other shrews trapped in the same study areas, Banyuls/Cerbère and Corsica. Specifically, in 22 out of 69 *C. russula* analyzed in Banyuls/Cerbère, 31.88% harbored intestinal trematodes belonging to *Brachylaima* (30.43%) and *Pseudoleucochloridium* (2.89%) genus, and 5.26% of the 95 *C. suaveolens* from Corsica harbored also *Brachylaima* sp. [40]. These terrestrial species have three-host life cycles with snails as first and second intermediate hosts. Although shedding light on the nutritional habits of *S. etruscus* in nature is difficult, Dr Fons supplied various mollusks, such as snails (*Rumina decollata*, *Helicella* sp., *Helix* sp.) and slugs (*Testacella* sp.) to the S. *etruscus* he kept in captivity, observing that the shrews tend to bite the mollusks which release mucus. The Etruscan shrews were satisfied by just licking the mucus; further attack on the snails being unusual [18]. Therefore, apparently, snails are not normally ingested by *S. etruscus*, thus making the presence of these trematodes in its helminth community rather unlikely.

Regarding the absence of acanthocephalans, we have to consider that the number of acanthocephalan species in small mammals is very low when compared to other helminths [69]. In fact, in 1026 shrews (662 *C. russula*, 171 *C. suaveolens*, 26 *Crocidura* sp., and 166 *S. etruscus*) studied in different locations of Spain and France, we only found a larval stage of *Centrorhynchus appendiculatus* in one individual of *C. russula* from the island of Ibiza [40,44]. Therefore, the lack of representatives of acanthocephalans in *S. etruscus* is not an unexpected phenomenon.

Shrews act as paratenic host in the two-host life cycle of acanthocephalans. The mammalian definitive host sheds the eggs that are ingested by different insects in terrestrial life cycles. In the intermediate host, the parasite goes through several developmental larval stages until it reaches the infective stage (cystacanth). When small mammals ingest the infected insects, the larval stage is encapsulated in the body cavity or muscles.

An additional fact that makes the presence of acanthocephalan larval stages in *S. etruscus* difficult is the nature of the insect intermediate host. The *S. etruscus* diet consists mainly of arthropods, with the exception of certain species: those chemically protected (like *Graphosoma*), too strongly chitinized like various Tenebrionidae beetles (*Blaps*, *Scaurus*, etc.); and those exceeding a certain size (like large Orthoptera) [18]. Laboratory studies on acanthocephalan life cycles typically used *Tenebrio molitor* as intermediate host since it is well-known that acanthocephalans with terrestrial life cycles usually infect well chitinized insects, especially Coleoptera and Orthoptera, thus making its presence, particularly in *S. etruscus,* difficult.

We would like to highlight the very low prevalence of adult nematode species, only 1.20% (Table 1), among the individuals of *S. etruscus* analyzed. However, 50 out of the 69 (72.46%) *C. russula* studied in the same Banyuls/Cerbère area harbored adult nematodes (unpublished data). Several species of nematodes were found in the esophagus, stomach, intestine, respiratory system, bladder, spleen, and liver of the greater white-toothed shrew. The reasons why, apparently, nematodes have not been able to colonize and/or adapt to *S. etruscus* pose a complex issue, considering that Nematoda is the most speciose phylum after Arthropoda, with about 12,000 species in vertebrates only [70]. At microevolutionary scale, host ecology and physiology have a strong influence on the evolution of host–parasite interactions, with both factors acting as ‘filters’ [71]. Like all mammals, *S. etruscus* is considered homeothermic; however, Etruscan shrews can enter a state of torpor to save energy when experiencing cold stress or food restriction [72]. Body temperature can fall as low as 6 °C. According to our results, the particular physiology of the smallest living mammal could have a negative influence, particularly, on its nematode fauna.

Considering data on the parasite species richness of other shrews of the *Suncus* genus, there are only information on *S. murinus* and *S. varilla minor*. The helminth community of the Asian house shrew, *S. murinus*, a commensal species that can weigh up to 150 g and measure 10–15 cm, is well-known and comprises more than 60 parasite species, specifically, four trematodes, almost 40 cestodes, and about 20 nematode species [57], as a result of hundreds of individuals analyzed. However, 39 individuals of this shrew analyzed in Cambodia harbored only five species [57], which clearly reinforces the positive correlation between sample size and parasite richness. Only two individuals of the African lesser dwarf shrew, *S. varilla minor*, have been parasitologically analyzed so far. Only one (new) tapeworm species has been reported in one of the two shrews studied, specifically the intestinal hymenolepidid *Rodentolepis gnoskei* Greiman and Tkach, 2012 [73], apparently specific of this shrew.

No specimens of *S. etruscus* have been studied in the same locations in which the Etruscan shrew coexists with *S. murinus* or *S. varilla minor*, to ascertain the specificity of *S. etruscus* helminth community. However, no adult helminth species detected in *S. etruscus* has ever been reported neither in any other shrew nor in any other small mammal, which suggests, regardless of their richness, the specificity of the *S. etruscus* helminth community, in particular of its cestode fauna. This result is consistent with the current knowledge on the specificity of cestodes parasitizing insectivorous mammals and this is particularly characteristic of shrew cestodes of the family Hymenolepididae, which are preferably mesostenoxenous (parasites which have more than one host species but restricted to one genus [74]). However, in the case of *S. etruscus*, its cestodes seem to have a higher degree of specificity, i.e., they are oioxenous (parasites that have only one host species [74]).

### 4.3. Helminth Diversity and S. etruscus Body

Mammalian orders vary greatly in the number of parasite species they harbor which is true for both endo- and ectoparasites. As the host body is the habitat for endoparasites, variation in body characteristics such as body size, metabolic rate, and longevity have often been considered determinant factors of parasite diversity [75]. *S. etruscus* has the smallest body size among mammals, a high oxygen consumption (while resting 67 times as much as resting humans) [28], and a short life span (16–18 months). The impact these intrinsic factors may have on the helminth diversity of the smallest living mammal deserves evaluation. However, we are aware of the fact that most of the *S. etruscus* analyzed in this study come from a single geographical area (Banyuls/Cerbère), while *S. etruscus* has a wide geographical distribution around the world, as pointed out in the Introduction section. This fact considerably limits the interpretation of the results obtained herein since other essential elements, also determinants of the helminth richness such as extrinsic factors (quality and diversity of habitat, season, etc.), as well as the analysis of the results according to the host population densities (host population density is one of the most important factors influencing the spread and distribution of parasites among host individuals [76]), could not be considered in this study.

It has been suggested that small-bodied mammals present a higher parasite diversity than large-bodied mammals. However, whilst some studies reported a positive correlation between parasite species richness and body size in mammals, other studies did not find this correlation [75]. According to our results, only seven parasite species were detected, and most of the individuals of *S. etruscus* analyzed (64.29%) harbored only one parasite species. However, the lack of helminthological results in other very small mammals such as the Bumblebee bat (*Craseonycteris thonglongyai*) or the Pygmy jerboa (*Salpingotulus michaelis*) do not allow to ascertain the real extent of the influence of the body size on the richness of the helminth communities.

Concerning the metabolic rate, its influence on parasite richness is also controversial [75]. It was hypothesized that high host metabolic rates could increase the probability of acquisition of parasites through increased food intake [77]. *S. etruscus* has a high basal metabolic rate reflective of their high surface area-to-volume ratio. They cannot survive for more than a few hours without food, and they must consume as much as six times their body weight in crickets and other insects on a daily basis [78]. This high food intake could increase the probability of parasite acquisition through the ingestion of the arthropod intermediate hosts. However, according to our results, this high metabolic rate and food intake of *S. etruscus* do not seem to exert a significant influence on the acquisition of parasites.

In general, the relationship between host species longevity and parasite diversity is poorly known. It was pointed out that parasite species richness was negatively correlated with host longevity independent of body mass [79]. In general, regardless of the species, shrews usually live between one to two years. Therefore, no conclusion can be reached on the influence of longevity in parasite diversity since *S. etruscus* has a similar lifespan as other shrew species which exhibit considerable higher parasite richness [17].

## 5. Conclusions

No data on the helminth community of the smallest living mammal in the world had been available until the 1980s when the descriptions of the three new species of intestinal cestodes were published [31,32,33]. Herein, the only study considering the global helminth spectrum of *S. etruscus* has been presented.

Although further molecular studies are required to ascertain the taxonomic status of the tapeworm of the genus *Pseudhymenolepis* found, current evidence seems to indicate that it is specific of *S. etruscus*, like the remainder of its adult cestode fauna.

Concerning the finding of two individuals of the genus *Anchotheca* in the stomach of the Etruscan shrew, obtaining new material is essential to carry out the specific identification. However, taking into account both the problematic trapping of *S. etruscus* as well as the considerably low prevalence of the nematode herein reported (1.33%, Table 2), it seems that the specific identification of the nematode is going to remain an open question in the foreseeable future.

According to our results, with the exception of the extraintestinal metacestodes reported, the helminth community of *S. etruscus* seems to be highly specific, i.e., oioxenous, and closely related to its insectivore diet.

Due to its small size, *S. etruscus* has undergone numerous physiological adaptations that have probably influenced its helminth spectrum, that is, the cestode specificity, the lack of trematodes and acanthocephalans, as well as the poorness of its nematode fauna. At any rate, and as it happens with any other correlation involving a host trait and parasite diversity, more than one process is probably involved, and all of these processes have contributed to shaping the observed helminth community pattern. Consequently, not only intrinsic factors of *S. etruscus*, but also the already mentioned extrinsic ones (host population densities, quality and diversity of habitat, season, environmental conditions, etc.) should be analyzed. Herein, the insufficient host sample size prevented a statistical study, making this type of analysis impossible.

In order to definitely characterize the *S. etruscus* helminth community, a larger host sample size should be obtained from different zones of its geographical distribution. However, the difficulty of trapping this minuscule mammal—several times highlighted—makes obtaining a significative number of individuals from different localities, rather unlikely. Therefore, notwithstanding the limitations we have faced in terms of material availability, we consider these first data on the helminth community of the smallest living mammal on Earth remarkable.

## Figures and Tables

**Table 1:** Helminth community of the 166 *Suncus etruscus* studied.

**Helminth Species**	**Microhabitat**	**n**	***p* (%)**	**95% CI**
*Mesocestoides* sp. *larvae*	abdominal cavity	1	0.60	0.10–3.96
*Joyeuxiella pasqualei larvae*	liver	2	1.20	0.10-3.96
*Staphylocystis claudevaucheri*	intestine	31	18.67	13.42–25.72
*Staphylocystis cerberensis*	intestine	5	3.01	1.01–6.85
*Staphylocystis banyulsensis*	intestine	14	8.43	4.46–13.13
Hymenolepididae gen. sp. indet.	intestine	9	5.42	2.27–9.47
*Pseudhymenolepis* sp.	intestine	47	28.31	21.42–35.39
**Overall cestodes**		80	48.19	40.30–55.78
*Aonchotheca* sp.	stomach	2	1.20	0.10–3.96
Nematoda gen. sp. *larvae*	stomach/abdominal cavity	6	3.61	1.61–8.18
**Overall nematodes**		8	4.82	2.27–9.47
**Overall parasitism prevalence**		84	50.60	43.00–58.21

Abbreviations: n, number of parasitized *S. etruscus*; *p*, prevalence; CI, confidence interval.

**Table 2:** Helminth community of *Suncus etruscus* according to the studied areas.

**Helminth Species**	**B/C (N = 150)** **n/*p* (%)**	**95% CI**	**Corsica (N = 16)** **n/*p* (%)**	**95% CI**
*Mesocestoides* sp. *larvae*	-	-	1/6.25	0.16–30.00
*Joyeuxiella pasqualei larvae*	2/1.33	0.01–4.20	-	-
*Staphylocystis claudevaucheri*	29/19.33	13.17–26.10	2/12.50	1.56–37.96
*Staphylocystis cerberensis*	3/2.00	0.45–5.71	2/12.50	1.56–37.96
*Staphylocystis banyulsensis*	14/8.43	4.30–13.46	-	-
Hymenolepididae gen. sp. indet.	7/4.67	2.17–9.76	-	-
*Pseudhymenolepis* sp.	43/28.67	22.00–36.85	4/25.00	7.31–51.69
*Aonchotheca* sp.	2/1.33	0.01–4.20	-	-
Nematoda gen. sp. *larvae*	6/4.00	1.53–8.46	-	-
**Overall prevalence**	78/52.00	44.00–60.00	6/37.50	15.31–63.51

Abbreviations: B/C, Banyuls/Cerbère; N, number of *S. etruscus* studied; n, number of parasitized *S. etruscus*; *p*, prevalence; CI, confidence interval.

## Data Availability

Not applicable.
